# Clinical outcomes of advanced non-small-cell lung cancer patients with *EGFR* mutation, *ALK* rearrangement and *EGFR*/*ALK* co-alterations

**DOI:** 10.18632/oncotarget.11218

**Published:** 2016-08-11

**Authors:** Na-Na Lou, Xu-Chao Zhang, Hua-Jun Chen, Qing Zhou, Li-Xu Yan, Zhi Xie, Jian Su, Zhi-Hong Chen, Hai-Yan Tu, Hong-Hong Yan, Zhen Wang, Chong-Rui Xu, Ben-Yuan Jiang, Bin-Chao Wang, Xiao-Yan Bai, Wen-Zhao Zhong, Yi-Long Wu, Jin-Ji Yang

**Affiliations:** ^1^ Southern Medical University, Guangzhou, 510515, China; ^2^ Guangdong Lung Cancer Institute, Guangdong General Hospital & Guangdong Academy of Medical Sciences, Guangzhou, 510080, China; ^3^ Medical Research Center, Guangdong General Hospital & Guangdong Academy of Medical Sciences, Guangzhou, 510080, China; ^4^ Department of Pathology, Guangdong General Hospital & Guangdong Academy of Medical Sciences, Guangzhou, 510080, China

**Keywords:** non-small-cell lung cancer (NSCLC), epidermal growth factor receptor (EGFR), anaplastic lymphoma kinase (ALK), overall survival, cohort study

## Abstract

The co-occurrence of epidermal growth factor receptor (*EGFR*) mutations and anaplastic lymphoma kinase (*ALK*) rearrangements constitutes a rare molecular subtype of non-small-cell lung cancer (NSCLC). Herein, we assessed the clinical outcomes and incidence of acquired resistance to tyrosine kinase inhibitors (TKIs) in this subtype. So we enrolled 118 advanced NSCLC treated with TKIs. *EGFR* mutations and *ALK* rearrangements were detected by DNA sequencing or Scorpion amplification refractory mutation system and fluorescence in situ hybridization respectively. Immunohistochemistry was used to evaluate the activation of associated proteins. We found that nine in ten patients with *EGFR/ALK* co-alterations had good response with first-line EGFR TKI, and the objective response rate (ORR) of EGFR TKIs was 80% (8/10) for *EGFR/ALK* co-altered and 65.5% (55/84) for *EGFR*-mutant (P = 0.57), with a median progression-free survival (PFS) of 11.2 and 13.2 months, (hazard ratio [HR]=0.95, 95% [CI], 0.49-1.84, P= 0.87). ORR of crizotinib was 40% (2/5) for *EGFR/ALK* co-altered and 73.9% (17/23) for *ALK*-rearranged (P= 0.29), with a median PFS of 1.9 and 6.9 months (hazard ratio [HR], 0.40; 95% [CI] 0.15-1.10, P = 0.08). The median overall survival (OS) was 21.3, 23.7, and 18.5 months in *EGFR*-mutant, *ALK*-rearranged, and *EGFR*/*ALK* co-altered (P= 0.06), and there existed a statistically significant difference in OS between *ALK*-rearranged and *EGFR*/*ALK co-altered* (P=0.03). Taken together, the first-line EGFR-TKI might be the reasonable care for advanced NSCLC harbouring *EGFR*/*ALK* co-alterations, whether or nor to use sequential crizotinib should be guided by the status of *ALK* rearrangement and the relative level of phospho-EGFR and phospho-ALK.

## INTRODUCTION

**Figure 1:**
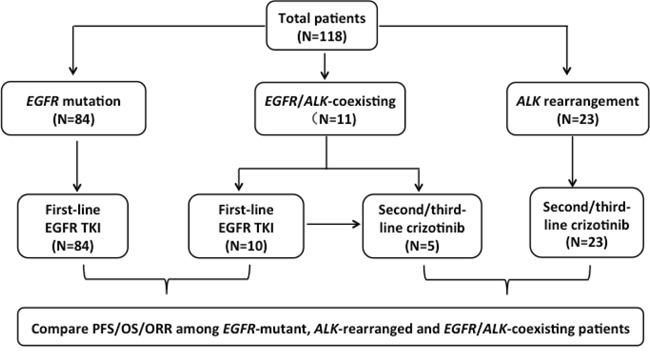
The design flow of this cohort study The three study populations and subsets analyzed are shown. TKIs, tyrosine kinase inhibitors. PFS, progression-free survival. OS, overall survival. ORR, objective response rate.EGFR, epidermal growth factor receptor.ALK, anaplastic lymphoma kinase.

Lung cancer is the leading cause of cancer-related death in the world [1]. However, recent discovery of oncogenic drivers has transformed the care of lung adenocarcinomas dramatically [2]. *Epidermal growth factor receptor (EGFR),* and *anaplastic lymphoma kinase (ALK),* are important oncogenic drivers in non-small-cell lung cancer (NSCLC) [2], and other oncogenic drivers in NSCLC, such as *ROS1* and *KRAS*, have also been found [3, 4]. Advances in targeted therapeutic agents have already approved for the treatment of patients with EGFR mutation and ALK translocation [5]. Early studies suggested that *EGFR* mutations and *ALK* rearrangements are mutually exclusive [6]. However, isolated cases with concomitant *EGFR* and *ALK* alterations have been reported [7–20], and the co-occurrence of multiple oncogenic drivers was also published [21, 22]. These two oncogenic drivers might overlap, and little has been known on both molecular biology and the role of EGFR or ALK inhibitors in such EGFR/ALK co-altered NSCLC. The prevalence of *EGFR*/*ALK* co-alterations was different [17, 18, 20, 23, 24], and it increased when more sensitive methods of detection (next-generation sequencing, NGS) were applied [24]. The responses to EGFR and/or ALK inhibitors were varied [9, 11, 16–18, 20, 24]. One study showed that the frequency of *EGFR/ALK* co-alterations was 1.3% (13/977) in NSCLC [20]. Beyond evidence of co-occurrence, the efficacy of EGFR tyrosine kinase inhibitors (TKIs) and ALK inhibitors has been revealed to be predicted by the relative levels of phospho-EGFR and phospho-ALK [20]. However, little has been known about the overall survival (OS) and molecular mechanisms of resistance to EGFR or ALK inhibitors in *EGFR/ALK* co-altered NSCLC. To address these issues, we performed a retrospective investigation in a cohort of *EGFR* mutation, *ALK* rearrangement and *EGFR/ALK* co-altered patients. The design flow of this cohort study was seen in Figure 1.

## RESULTS

### Patient characteristics

In our previous study (2009-2011) of 11 *EGFR*/*ALK* co-altered patients treated with EGFR and/or ALK inhibitors, only objective response rate (ORR) and progression-free survival (PFS) were analyzed. And now, we aimed to investigate OS, the potential mechanisms of resistance to TKIs and the appropriate regimen for *EGFR*/*ALK* co-altered patients. As a comparison, totally, 84 advanced NSCLC patients with *EGFR* mutations treated with first-line EGFR TKIs, and 23 with only *ALK* rearrangements treated with second or further-line crizotinib, were retrospectively enrolled to investigate OS in the present study during the same period of time. From August 2009 to July 2011, 118 NSCLC patients treated with TKIs were enrolled, and the clinical characteristics of them were listed in Table 1. All patients were of Chinese ethnicity. The median ages were 63, 48 and 59 years for the *EGFR*-mutant, *ALK*-rearranged, and *EGFR/ALK* co-altered patients, respectively (P < 0.001).

**Table 1:** Baseline demographic and clinicopathologic characteristics in the three subgroups

Characteristic	*EGFR* mu (%)	*ALK* re (%)	*EGFR/ALK* (+) (%)	Total (%)	P
Gender					0.206
Male	33(39.3%)	13(56.5%)	3(27.3%)	49(41.5%)	
Female	51(60.7%)	10(43.5%)	8(72.7%)	69(58.5%)	
Age (median)	63(38-85)	48(26-75)	59(40-71)	60.5(26-85)	<0.001^*^
WHO PS					0.061
0-1	81(96.4%)	19(82.6%)	10(90.9%)	110(93.2%)	
2-3	3(3.6%)	4(17.4%)	1(9.1%)	8(6.8%)	
Smoking history					0.377
Non smoker	69(82.1%)	20(87.0%)	11(100.0%)	100(84.7%)	
Heavy smoker	15(17.9%)	3(13.0%)	0(0.0%)	18(15.3%)	
Histology					1.000
AC	82(97.6%)	23(100%)	11(100.0%)	116(98.3%)	
SCC	2(2.4%)	0(0.0%)	0(0.0%)	2(1.7%)	
Clinical stage					0.343
III	2(2.4%)	0(0.0%)	1(9.1%)	3(2.5%)	
IV	82(97.6%)	23(100.0%)	10(90.9%)	115(97.5%)	
Total	84(71.2%)	23(19.5%)	11(9.3%)	118(100.0%)	

Aberrations: mu, mutation; re, rearrangement; WHO PS, World Health Organization performance status; SCC, squamous cell carcinoma; AC, adenocarcinoma. *EGFR*, epidermal growth factor receptor; *ALK*, anaplastic lymphoma kinase; A heavy smoker was defined as a patient who had smoked >100 cigarettes in his or her lifetime or that had stopped smoking less than 15 years and had a total of ≥10 pack-year of smoking.

* The age in three groups was analyzed with the use of Wilcoxon test

Beyond this difference, the three groups were well balanced with respect to demographic and baseline characteristics. The majority of patients (84.7%) were never-smokers, and the most common histological subtype was adenocarcinoma (98.3%).

### Efficacy of TKI treatment

All patients were assessed for a TKI response; a detailed list of the treatments for each of the eleven patients with concomitant *EGFR* mutations and *ALK* rearrangements was given in Figure 1. The ORR of EGFR TKIs was 80% (8/10) for *EGFR/ALK* co-altered patients and 65.5% (55/84) for *EGFR*-mutant patients, respectively; this difference was not statistically significant (P= 0.57). Similarly, no statistical difference in the ORR to crizotinib between the *EGFR/ALK* co-altered (2/5; 40.0%) and *ALK*-positive (17/23; 73.9%) patients (P= 0.29).

**Figure 2:**
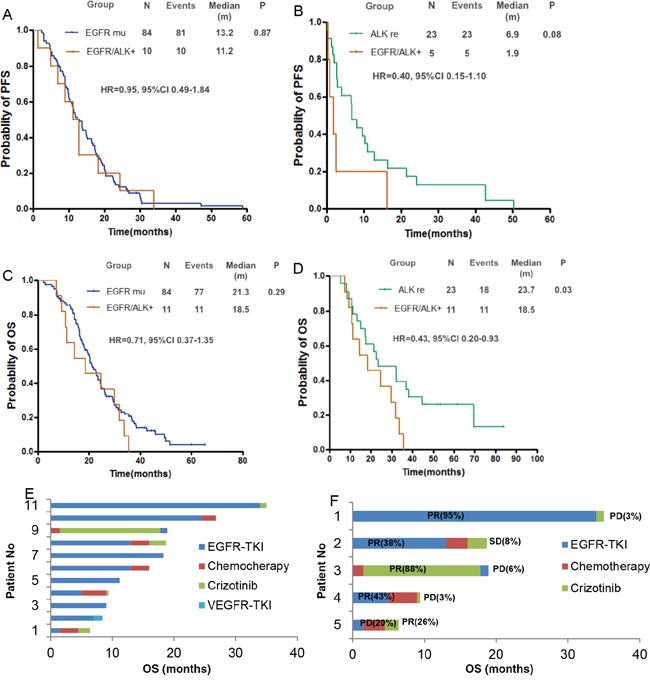
Comparisons of survival among the three groups **A.** Kaplan-Meier curves for PFS of *EGFR*-mutant patients and *EGFR/ALK*-coexisting patients **B.** PFS curves of *ALK*-rearranged patients and *EGFR/ALK*-coexisting patients. **C.** Kaplan-Meier curves for OS of *EGFR*-mutant patients and *EGFR/ALK*-coexisting patients **D.** OS curves of *ALK*-rearranged patients and *EGFR/ALK*-coexisting patients **E.** The contributions of chemotherapy or TKIs to OS for 11 *EGFR/ALK*-coexisting patients **F.** PFS of two TKIs contributing to OS for the five patients who received both ALK and EGFR TKIs. VEGFR, vascular endothelial growth factor receptor. Chemo, chemotherapy. PR, partial response. SD, stable disease. PD, progressive disease. ALK, anaplastic lymphoma kinase. EGFR,epidermal growth factor receptor. mu, mutation. re, rearrangement. PFS, progression-free survival. OS, overall survival. TKI, tyrosine kinase inhibitor.

The last follow-up time was July 13 2015, and the median follow-up time was 23.2 months. The median PFS was 13.2 months in *EGFR*-mutant patients and 11.2 months in *EGFR/ALK* co-altered patients treated with EGFR TKIs (hazard ratio [HR], 0.95; 95% confidence interval [CI], 0.49-1.84; P = 0.87) (Figure 2A). The median PFS was 6.9 months for *ALK*-rearranged patients and 1.9 months for *EGFR/ALK* co-altered patients treated with crizotinib (HR, 0.40; 95% CI, 0.15 - 1.10;P= 0.08) (Figure 2B). The median OS were 21.3, 23.7, and 18.5 months for *EGFR*-mutant, *ALK*-rearranged, and *EGFR/ALK* co-altered patients, respectively (P = 0.06). The OS of subgroup of *EGFR*-mutant and *EGFR*/*ALK* co-altered patients did not have statistically significant difference (HR, 0.71; 95% CI, 0.37-1.35;P=0.29) (Figure 2C), but the statistical difference between *ALK*-rearranged and *EGFR*/*ALK* co-altered was significant (HR, 0.43; 95% CI, 0.20 - 0.93; P=0.03) (Figure 2D). Among 11 *EGFR/ALK* co-altered patients, all received EGFR TKI, and ten received EGFR TKI as the first line, and nine of them had good response and prolonged survival, but the last one had progression disease and shorter PFS. One with second-line crizotinib had a good response; after progression, EGFR TKI was taken without good response and the tendency of PFS was shorter (Figure 2E). Five patients received both TKIs, four with first-line EGFR TKI, and three of them had clinical benefits and prolonged survival, but without benefits from the following TKI, the same as the one taking ALK TKI and subsequent EGFR TKI (Figure 2F).

### Potential mechanism of TKI resistance

**Figure 3:**
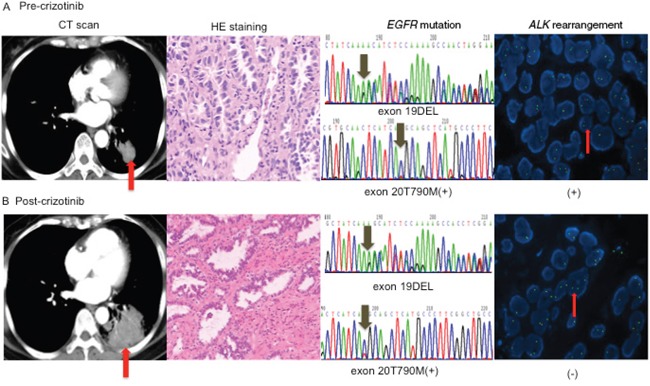
Changes of driver genes in *EGFR/ALK*-coexisting patient treated with crizotinib **A.** Pre-crizotinib histology was adenocarcinoma (original magnification×200), and genetic profiles showed *EGFR* exon 19 deletion and exon 20 T790M mutation detected by DNA sequencing, as well as positive *ALK* rearrangement detected by FISH **B.** After resistance to crizotinib, both the histology (original magnification×200) and *EGFR* mutation were the same as the baseline, but *ALK* rearrangement was negative. *ALK*, anaplastic lymphoma kinase. *EGFR*, epidermal growth factor. DEL, deletion.

**Figure 4:**
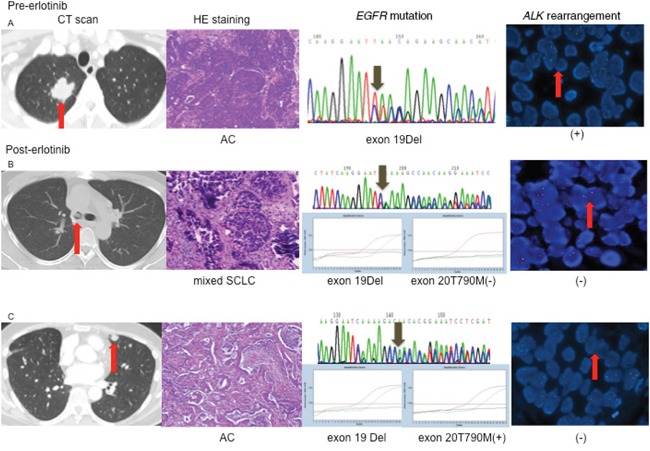
Changes of driver genes in different lesions with EGFR-TKI **A.** Before EGFR-TKI treatment, the pathology of lung lesion was adenocarcinoma, *EGFR* mutation detected by sequencing was exon 19 deletion, and *ALK* was positive **B.** After EGFR-TKI treatment, the pathology of the right main bronchus was mixed small-cell lung cancer with adenocarcinoma; *EGFR* mutation detected by ARMS was exon 19 deletion. *ALK* rearrangement was negative **C.** the pathology of the lesion in the left upper lung lobe was adenocarcinoma, *EGFR* mutation was exon 19 deletion and exon 20 T790M mutation. AC, adenocarcinoma. *ALK*, anaplastic lymphoma kinase. DEL, deletion.*EGFR*, epidermal growth factor receptor.SCLC, small-cell lung cancer.

**Figure 5:**
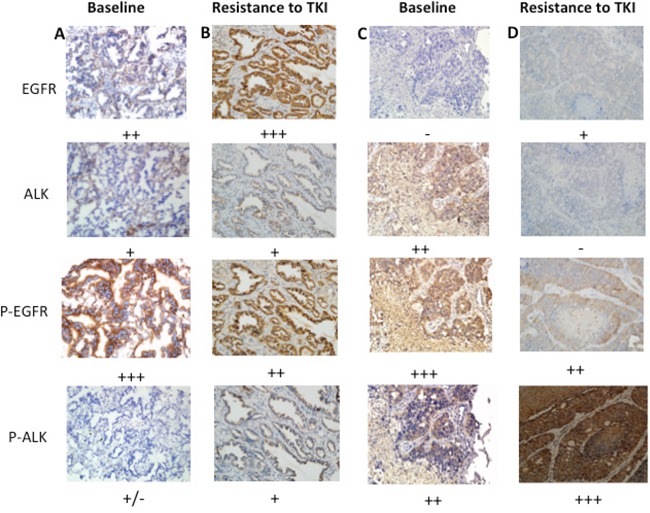
Dynamic changes of expression of protein in EGFR/ALK co-existing patients who received TKI **A.** After resistance to erlotinib, P-EGFR was at higher level than P-ALK. **B.** After resistance to crizotinib, P-EGFR was higher than P-ALK. **C.** Pre-EGFR-TKI, P-EGFR was higher than P-ALK. **D.** After resistance to erlotinib, the level of P-ALK was higher than P-EGFR. P-, phospho-. ALK, anaplastic lymphoma kinase. EGFR, epidermal growth factor receptor.TKI, tyrosine kinase inhibitor.

The potential mechanisms of resistance to TKIs in the two *EGFR/ALK* co-altered patients were shown in Figure 3 and Figure 4. The first case received first-line erlotinib for nine months, achieving a partial response; however, after tumour progression, she was switched to intercalated erlotinib and chemotherapy for eight months, achieving stable disease. The patient then received crizotinib, and progressive disease occurred two months later, and re-biopsy was performed. Her genetic profiles were as follows: *EGFR* mutation with both an exon 19 deletion and exon 20 T790M mutation, negative *ALK* rearrangement (Figure 3). The level of phospho-EGFR decreased, while expression of phospho-ALK was increased, and the level of phospho-EGFR was higher than phospho-ALK after resistance to crizotinib (Figure 5A, 5B). *EGFR* exon 20 T790M mutation had been linked to resistance to erlotinib, and relative higher level of phospho-EGFR than phospho-ALK might be the result of resistance to crizotinib.

The second patient received erlotinib for thirteen months, exhibiting gradual progression without any symptoms; therefore, he continued on erlotinib for an additional seven months until dramatic progression. CT scan showed new lesions in the left upper lung lobe and the right main bronchus, and wedge resection of the left upper lobe revealed a lung adenocarcinoma harbouring an *EGFR* exon 19 deletion and exon 20 T790M mutation. Two weeks later, he received bronchoscopy, and the result showed small-cell lung cancer (SCLC) mixed with an adenocarcinoma harbouring an *EGFR* exon 19 deletion. The two lesions were both negative for *ALK* rearrangements after treatment with erlotinib (Figure 4). Phospho-EGFR was decreased from its initial high level, but the level of phospho-ALK was higher than pre-treatment of erlotinib, and the level of phospho-ALK was higher than phospho-EGFR after resistance to erlotinib (Figure 5C, 5D).

Taken together, these data suggested the relative higher level of phospho-EGFR was the mechanism of resistance to crizotinib; the *EGFR* exon 20 T790M mutations, pathology transformation from adenocarcinoma to SCLC and the relative higher level of phospho-ALK as molecular mechanisms of resistance to EGFR TKIs.

## DISCUSSION

Herein, we analysed OS in advanced NSCLC patients harbouring concomitant *EGFR* mutations and *ALK* rearrangements, as well as the potential mechanisms underlying acquired resistance to EGFR-TKI and/or crizotinib in two cases.

To *EGFR/ALK* co-altered patients with sequential both EGFR TKI and ALK TKI, EGFR TKI had good response and prolonged survival for most of them, but primary resistance occurred to the subsequent ALK TKI. It seemed that first-line EGFR TKI was a reasonable regimen for most of the subgroup, but single ALK TKI was excluded from the following eligible treatment. They exhibited a tendency towards relatively shorter OS than those with either an *EGFR* mutation or *ALK* rearrangement, and there existed statistically significant difference between the subgroup of *ALK* rearrangement and *EGFR/ALK* co-alterations. This difference should be related to the different first/second-line therapeutic regimens administered to these subgroups. Crizotinib is a standard of care for ALK-rearranged patients, and it can prolong survival, but as to EGFR/ALK co-altered patients in our study, crizotinb was only used as second- or further-line therapy and might not favor efficacy and survival. On the other hand, efficacy of crizotinib might be related to the abundance of ALK rearrangement determined by the sensitivity of test method. As to the first case with double TKIs, after resistance to EGFR TKI and chemotherapy, the level of phospho-EGFR was higher than phospho-ALK, although ALK rearrangement was positive, primary resistance to crizotinib occurred, and at that time the level of phospho-EGFR was still higher than phospho-ALK. The second case only receiving EGFR TKI had negative ALK rearrangement after resistance to EGFR TKI. The lack of efficacy of crizotinib in the subgroup of EGFR/ALK co-altered patients could be correlated with the lower activation of ALK than EGFR signaling pathway or relatively lower abundance of ALK rearrangement after resistance to EGFR-TKI and/or chemotherapy. A re-biopsy for the detection of EGFR mutation and ALK rearrangement would be necessary to select the appropriate therapeutic regimen for these patients. The second case only receiving EGFR TKI had negative ALK rearrangement after resistance to EGFR TKI, and the loss of ALK rearrangement might correlate with EGFR TKI. Previous research showed that one driver gene may be sufficient to drive resistance to TKIs in dual EGFR/ALK co-altered NSCLC [11]. Combination of both TKIs may be the potential choice after progression with first-line EGFR-TKI.

The result of the study demonstrated that first-line EGFR TKI might be the reasonable care for advanced NSCLC patients harboring concomitant *EGFR* mutations and *ALK* rearrangements, but the result of the other study was conflicting, suggesting that ALK inhibitors appeared to be effective for patients with *EGFR*/*ALK* co-alterations [24]. In our study, we detected EGFR mutation with DNA direct-sequencing, and in the other study, a more sensitive test method, called as the next-generation sequencing, was applied [24]. The abundance and the intratumoral heterogeneity of *EGFR* mutation and *ALK* rearrangement probably correlated with the efficacy of EGFR TKI and/or ALK TKI in EGFR/ALK co-altered patients [25]. The diagnosis of driver gene status might be affected by the sensitivity of detection methods, and therapy selection may make tumors become more heterogeneous for intratumoral genetic heterogeneity, which might be the major reason for resistance to TKIs [26]. As to *EGFR*/*ALK* co-altered patients, both oncogenic drivers status should be evaluated for the appropriate management of patients using more sensitive assays. The abundance of *EGFR* mutation and *ALK* rearrangement and the levels of phosphorylation of downstream proteins are detected, and treatment strategies might be optimized for patients with concomitant *EGFR* and *ALK* alterations in the future.

Despite the favourable efficacy of TKIs in patients with *EGFR* mutations or *ALK* rearrangements, resistance to these drugs is inevitable. Both *EGFR*-mutant and *ALK*-rearranged lung cancers utilise common mechanisms of resistance, including the development of secondary mutations in the tyrosine kinase domain, driver gene amplification, phenotypic alterations, and reactivation of the primary signalling pathways through alternative signalling molecules [27–29]. Of these many different mechanisms of resistance, approximately half of all lung carcinomas with acquired resistance to EGFR TKIs develop a T790M point mutation in exon 20 of *EGFR* [30, 31]. The T790M mutation is thought to reactivate EGFR signaling by increasing the receptor's affinity for ATP over TKIs [32] This mutation is also thought to amplify the expression of components of the hepatocyte growth factor receptor and mesenchymal epithelial transition (MET) pathways [27, 33–36] In addition, *PIK3CA* and *BRAF* mutations, as well as tumour morphological changes such as transformation from NSCLC to SCLC small cell lung cancer and epithelial to mesenchymal transitions have been observed, though the mechanisms by which they develop and lead to resistance are incompletely understood [27, 34, 37]. The mechanisms of acquired resistance to crizotinib in NSCLC include secondary mutations within the ALK kinase domain, amplification of the *ALK* gene, and bypass of important signalling mechanisms via an increase in EGFR phosphorylation or KIT amplification, and so on [23, 28, 38]. The frequency of *ALK* negative tumor was observed in patients with resistance to crizotinib was 18.2% [23], given that the emergence of an *ALK* negative tumor was associated with other separate oncogenic drivers, or the percentage of positive cells in this case was not zero, consistent with background noise in the break apart FISH assay as previously described [7]. Given the limited tumor sample of the first case, we were unable to detect the presence of other oncogenic drivers. Though the molecular mechanisms underlying acquired resistance to EGFR TKI and crizotinib in *EGFR/ALK* co-altered patients were similar to those in typical *EGFR* mutation and *ALK* rearrangement patients, such as *EGFR* exon 20 T790M mutation and pathologic transformation from adenocarcinoma to SCLC small-cell lung cancer have been linked to resistance to erlotinib. As well as the relative higher level of phospho-ALK than phospho-EGFR is related to resistance to EGFR TKI. In addition to phospho-EGFR is higher than phospho-ALK is also related to resistance to crizotinib. The mechanisms of acquired resistance in patients with more than one positive driver genes were inevitably more complex. As to *EGFR*/*ALK* co-alterations, status of *EGFR* mutations and *ALK* rearrangements should be known to us, the downstream proteins of the two pathways should also be detected, and we could have the eligible regimen for the special group through detection of dynamic changes of the downstream proteins of the two pathways.

The level of phosphorylation of downstream protein is related to the activation of the signaling pathway, and detection of the relative phosphorylation levels of EGFR and ALK might help to guide the selection of TKIs in clinical practice [20]. The dynamic change of the level of the phosphorylation, for example, increased or decreased, can be detected by IHC, and the variation of staining intensity might predict the efficacy of TKIs [20, 38]. As to the tumor with 2 oncogenic drivers, which agent becomes more effective might depend on the levels of the relevant gene alterations. Which signaling pathway is the dominant one should be investigated initially, and re-biopsy is very important to analyze the potential mechanisms of resistance to TKIs.

To the best of our knowledge, this is the first study examining OS and the molecular mechanism of resistance to EGFR or ALK TKI in *EGFR/ALK* co-altered patients. However, this study had a few limitations. First, this was a retrospective, single-institution study, which could not accurately reflect the population with *EGFR*/*ALK* co-alterations at large. Second, the numbers of phospho-EGFR and phospho-ALK tests were small. Finally, the lack of sufficient biopsy samples limited our investigation of the mechanisms underlying TKI resistance.

In summary, first-line EGFR TKI might be the reasonable care for advanced NSCLC patients harbouring concomitant *EGFR* mutations and *ALK* rearrangements. As to this special gene profile patients, the molecular mechanisms underlying acquired resistance to EGFR TKI or crizotinib were similar to those of *EGFR* mutation and ALK rearrangement patients, but whether or nor to use sequential or in combination with crizotinib should be guided by the status of ALK rearrangement and the relative level of phospho-EGFR and phospho-ALK. Additional investigations are needed to overcome resistance to EGFR or ALK TKI and to improve OS in patients harbouring *EGFR/ALK* co-alterations.

## MATERIALS AND METHODS

### Patients and treatment

Patients were eligible for inclusion in the study if they were 18 years old or older, had histologically or cytologically confirmed clinical stage IIIB or IV NSCLC with histologic feathers of adenocarcinoma or squamous cell carcinoma. All patients provided written informed consent; separate consent was provided for the assessment of EGFR and ALK biomarkers. An independent ethics committee at the institution approved the study protocol. The study was conducted in accordance with the Declaration of Helsinki, the International Conference on Harmonization Guidelines for Good Clinical Practice and applicable requirements. We recruited 118 patients from the Guangdong Lung Cancer Institute (GLCI) between August 2009 and July 2011. Among these patients, we identified 11 patients with concomitant *EGFR* mutations and *ALK* rearrangements treated with EGFR TKIs, crizotinib, or both, These *EGFR*/*ALK* co-altered patients were the same as those having evaluable clinical data as previously published [20]. Two additional groups, consisting of 23 *ALK*-positive patients receiving second or later-line crizotinib therapy as part of the A8081005 (NCT0093245) and A8081007 (NCT0093289) trials [39, 40], A total of 4 cases with *ALK* rearrangement also enrolled in our previous study [20]. and 84 *EGFR*-mutant patients enrolled in the CTONG 0901 trial receiving the first-line EGFR TKIs gefitinib and erlotinib [41] were used as controls, once progressive disease occurred, the further therapy after progression of the disease was at the physician's discretion.

### EGFR mutation analysis

Genetic profiles were identified in the Department of Pathology of GGH and the GLCI. All tumour samples were routinely assessed by sectioning, haematoxylin-eosin staining, and visualisation under a microscope to ensure a tumour content of at least 50%. A mutation analysis of the *EGFR* tyrosine kinase domain was performed by DNA directed sequencing and/or the amplification refractory mutation system (ARMS) as described previously [42, 43] and according to the manufacturer's protocol, patients were considered to be positive for the *EGFR* mutation if 1 of 29 *EGFR* mutations was detected with the above methods.

### RT-PCR and RACE-PCR sequencing for AK fusion analysis

Total RNA was extracted from tissue samples using the RNeasy Kit (Qiagen). Reverse-transcriptase PCR (RT-PCR) and 5′ rapid amplification c-DNA ends (RACE) coupled PCR plus sequencing was detected as described previously [8]. The products of PCR were then sequenced with a 3730XL Genetic Analyser (Applied Biosystems). Target sequences of interest were aligned with the ALK reference sequence (NM_004304.3) to determine whether the fusion with another gene appeared.

### Fluorescence in situ hybridization (FISH) assays for ALK rearrangements

Tumour histology was classified using World Health Organisation criteria. Interphase molecular cytogenetic studies were performed using a commercially available ALK probe (Vysis LSI ALK Dual Colour, Break Apart Rearrangement Probe; Abbott Molecular Inc.) using 4-μm thick paraffin-embedded tissue sections. Samples were deemed to FISH-positive, if > 15% of the scored tumour cells had split ALK 5′ and 3′ probe signals or isolated 3′ signals [44].

### Immunohistochemistry (IHC) for EGFR, ALK and downstream molecules

Protein expression was detected by IHC in serial sections of formalin-fixed paraffin-embedded tumour samples according to the manufacturer's instructions for each antibody. The samples were stained with antibodies against total EGFR, phospho-EGFR (Y1068), total ALK, and phospho-ALK (Y1604) (Cell Signalling Technology), followed by incubation in rabbit monoclonal anti-human ALK antibody (#3633 WP1-01; clone D5F3) at a dilution of 1:100. The staining intensity was scored from 0 to 3+; tumours with no expression (0) were described as negative for ALK protein expression, while tumours scored as 1+, 2+, or 3+ were deemed positive [45, 46], and the staining intensity was scored according to the following criteria: 3+, intense, granular cytoplasmic staining; 2+, moderate, smooth cytoplasmic staining; 1+, faint cytoplasmic staining in 10% of tumor cells; and 0, no staining [45], and high phosphorylation of proteins means IHC ++ or +++; low phosphorylation of proteins means IHC + or +/− NA, not available. -/NA, IHC negative because of not available testing [20, 38].

### Assessments and statistical analysis

Evaluation of efficacy was assessed every 6 to 8 weeks according to Response Evaluation Criteria in Solid Tumours (RECIST1.1) [47]. PFS was measured from the initial treatment with an EGFR TKI or crizotinib to radiographic or clinical progression, as determined by means of the RECIST1.1′s criteria, or death from any cause. OS was calculated from the date of first-line therapy to death from any cause, or the date of last follow-up. ORR was analysed by the χ^2^-test, and PFS and OS were analysed with the use of the Kaplan-Meier curves, and hazard ratios for progression or death were calculated using the Cox proportional-hazards model. Subgroup analyses were performed to compare PFS and OS between treatments in groups defined according to the WHO performance status, age, disease stage at screening, pathology and smoking status. Detection the status of biomarkers and treatment with covariates were used to identify predictive factors by assessing whether there was a significant difference in the biomarkers and treatment effect for PFS and OS (hazard ratio for progression or death) between subgroups. The χ^2^ test, Fisher's exact test, and Wilcoxon test were used to compare categorical variables. All statistical tests were two-sided, with P value < 0.05 considered statistically significant.
